# *“I Didn’t Realise I Knew That!”* Higher Education Students’ Experiences of Incidental Learning Through OpenWorld Role-Playing Games

**DOI:** 10.3390/bs16071123

**Published:** 2026-07-05

**Authors:** Rebecca Ferriday

**Affiliations:** School of Biosciences, Cardiff University, The Sir Martin Evans Building, Museum Avenue, Cardiff CF10 3AX, UK; ferridayr@cardiff.ac.uk

**Keywords:** incidental learning, OpenWorld Role-Playing Games, higher education, unintended consequences, Universal Design for Learning, principles of learning

## Abstract

OpenWorld Role-Playing Games (OWRPGs) differ from other video game genres in that they offer players vast digital landscapes and significant freedom: they can fight, use diplomacy, play stealthily, or pursue activities like farming or trading. This player autonomy creates a space rich in unintended consequences, incidental learning being just one. A study of higher education (HE) students explored this phenomenon. While existing research on incidental learning in games focuses mainly on language acquisition, the current study argues that commercial OWRPGs have the same educational power as purpose-built learning games, and that the scope of incidental learning is far broader than previously examined. Using a constructivist grounded theory (CGT) approach, 25 semi-structured interviews were conducted and written gaming journals collected from 11 additional participants. All 36 participants played OWRPGs in their leisure time. The analysis identified 34 distinct forms of incidental learning, with language acquisition being just one. These were grouped into three categories: *self-improvement*, *academic*, and *employability skills*, with considerable overlap between each. This study shows that OWRPGs can act as a setting for incidental learning in skills over and above lexical improvement. As a result, it is suggested that future research analyse in further depth the skills this research has discovered, looks for further skills that may be honed via incidental learning, and examine other video game genres to highlight any forms of incidental learning that occur.

## 1. Introduction

Individuals are surrounded by opportunities for learning, and the process of informal learning leads to the development of understanding often without meaning to. Marsick and Watkins (2001) state that while formal learning is usually institutionally sponsored, classroom-based, and highly structured, incidental learning is defined as a “byproduct of some other activity, such as task accomplishment, interpersonal interaction, and trial-and-error experimentation; three activities that play key roles in gaming experiences.” It can also be defined as “*Learning which apparently takes place without a specific motive or a specified formal instruction …*” (McGeoch, 1942), and links explicitly to the notion of intrinsic motivation: the undertaking of an activity for inherent satisfaction. Intrinsic motivation comes from within the individual, as opposed to extrinsic motivation, which comes from outside (Nickerson, 2023), and is a complementary dimension of Self-Determination Theory (SDT). SDT focuses on the innate psychological needs that drive human motivation and well-being and posits that individuals have three basic psychological needs: autonomy, competence and relatedness. The satisfaction of these needs is crucial for the development of intrinsic motivation (Ryan & Deci, 2000). Traditional education is generally orientated around extrinsic motivation: the student must learn what is provided by a curriculum by the teacher to pass or fail a course or assignment. Incidental learning is usually a byproduct of some other activity. It is spontaneous, unstructured, and learner evaluated (Marsick & Watkins, 1990). It is also more likely to be obtained if the information is related to a topic of interest, or in the case of this study, via a game:

*“The trick is not to teach the facts at all, but rather to have the facts be along the way to getting to something the student naturally wanted to know in the first place … we should use students′ natural interest, so they come across such facts incidentally, in the course of pursuing their interests.”*

(Schank, 1995)

Therefore, if the learner is motivated by doing something they find interesting, they can learn without noticing. It is this intrinsic motivation that is core to the incidental learning this study’s participants have spoken about: they were doing something in which they were immersed and that was interesting to them, while intrinsic motivation meant that they were learning informally. Unintended consequences underpin this informal learning; the unplanned, unregulated learning that occurs naturally through everyday interactions, which is a fundamental but often overlooked cornerstone of the learning process (Rutherford, 2017).

Incidental learning can also be said to be an unintended consequence.

It is important at this stage to highlight the etymological and sociological origin of the concept of the phrase “unintended consequences,” and how it has come to be used as an adage or idiomatic warning that an intervention in a complex system tends to create unanticipated and undesirable outcomes. Linked to this, the term is commonly used as a wry or humorous warning against the hubristic belief that humans can fully control the world around them. Yet despite this negative interpretation, the concept of unintended (or unanticipated) consequences was originally proposed by sociologist Merton (1936) as something that could be deemed as being positive, negative, or neutral.

An OpenWorld Role-Playing Game (OWRPG) is a video game that combines a vast, non-linear virtual environment with deep character progression. Players can freely explore the game world at their own pace and tackle objectives in any order, while improving their character’s skills and regularly making narrative choices. Such games offer players significant freedom: combat can be weapons or magic-based, or avoided completely by using diplomacy. Players can forgo combat by choosing a stealthy approach, evading enemies by moving silently through the shadows, or opting to pursue peaceful activities such as farming or trading. This player autonomy creates a space rich in unintended consequences, incidental learning being just one.

It was a personal experience that instigated this research into incidental learning as an unintended consequence of playing. Therefore, this research includes reference to my own experience and contains strong elements of personal insight.

**Figure 1:**
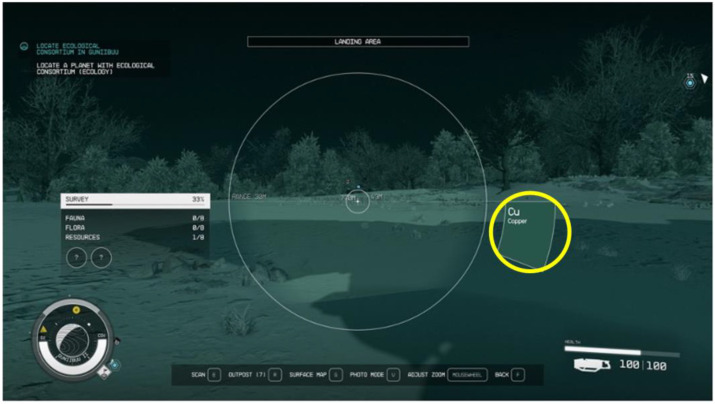
Using a scanning device in “Starfield” to locate copper (Cu), circled in yellow.

It was while watching a televised quiz show that I was surprised to discover that I was able to correctly answer one question requiring the identification of minerals represented as symbols from the periodic table of elements. I have never been a student of science, so was surprised to discover that I knew the names of these minerals. I realised that I had committed to memory much of the periodic table through playing a popular OWRPG: *Starfield* (Bethesda, 2023). I had not made a conscious decision to play this game with the express desire to learn about chemistry, but many hours spent scanning alien planets to find resources in-game suggested that I had learned, for all intents and purposes, by accident (see Figure 1). It was this appreciation that became the starting point of a study seeking to uncover the unintended consequences experienced by higher education students when playing digital OWRPGs.

However, while a wealth of research exists around lexical acquisition as incidental learning, and much research has been carried out around “purposeful learning,” often via games developed specifically for educative purposes, there is little research that examines other academic subject areas or skills that can be learned informally, and as a result of playing commercial video games—that is, games developed purely for recreational purposes. To begin to address this gap in the research, this paper identifies the impact of OWRPGs on skills acquisition, inclusive education, semiotics, Universal Design for Learning (UDL), and principles of learning.

## 2. Methods

The study used constructivist grounded theory (CGT) (Charmaz, 2014), a qualitative research methodology that positions the researcher as an active participant in constructing knowledge alongside their research participants. Rooted in interpretivism and social constructionism, CGT holds that reality is multiple, subjective, and socially constructed; that knowledge emerges from the interaction between researcher and participant; that findings are co-constructed rather than discovered; and that the researcher’s positionality, assumptions, and perspectives shape the inquiry.

Data was gathered through 25 interviews and 11 sets of gaming journals from undergraduate students who were regular players of OWRPGs, alongside a written journal of my own gaming experiences. A number of prompts were provided for journal-keepers, who were asked to record the name(s) of the game(s) they were playing, to think about the reasoning behind playing the game(s), to make a note of their mood at the start and end of the session, and to make a note of any unexpected experiences they had while they were playing. For parity across data, my gaming journal followed the same prompts. This information was then analysed simultaneously using the active, interpretive stance modelled through CGT.

All recorded interviews and journal entries were transcribed before implementing an initial coding phase using the incident-by-incident process common to CGT to attach short, active codes to incidents. Codes used gerunds (“managing gaming time,” “improving coordination”) to capture actions and processes, and after identifying significant and frequently appearing initial codes, focused coding started to reveal what was happening across the data. This second phase was a more conceptual process, with initial codes aggregated to these newly developed focused codes. Even at this early stage, there were signs of an emerging theory: that participants were honing a range of skills as an indirect result of playing OWRPGs.

From the start of the interview stage and through all phases of coding, memos were written to capture thoughts around codes, develop ideas, compare categories, and trace reasoning. Parallel to this, constant comparative analysis also occurred at every stage. Categories were further developed through focused coding and comparison and elevated into broader, more abstract categories with defined properties and dimensions. Once theoretical saturation was reached (that is, when data no longer generated new insights), memos and categories were reviewed for a final time to contribute to the construction of a coherent theoretical framework.

It became evident at the end of the coding process that incidental learning had taken place as a byproduct of playing OWRPGs for many participants, encompassing many subject areas and skills, and on many occasions.

## 3. Summary of Literature

Recent studies have explored the relationship between video games and incidental learning, particularly in the context of language acquisition. Of note is a 2021 study carried out by Calvo-Ferrer and Belda-Medina (2021) with secondary school students examining the effects of multiplayer video games on incidental and intentional vocabulary learning. The study found that while commercial games (that is, games developed expressly for pleasure, as opposed to “serious games” developed explicitly for educative purposes) can facilitate vocabulary acquisition, results varied, and further investigation has been recommended by the authors. In the same year, Qasim (2021) examined the impact of digital games on incidental vocabulary acquisition among Pakistani high school students. The research mapped out gaming habits, experiences, and perceptions regarding incidental English vocabulary acquisition, with a recommendation that future research employ vocabulary tests to further explore the conclusions drawn from the study. A separate study examining single-player games and lexical acquisition focused on how video games such as popular OWRPG *The Legend of Zelda: Breath of the Wild* (Nintendo Entertainment Planning and Development, 2017) impact second-language users’ incidental lexical learning. The case study involved Cantonese undergraduate students and assessed their vocabulary knowledge using the Vocabulary Knowledge Scale tests (Jung, 2022). Results indicated that playing the game improved participants’ receptive vocabulary knowledge, though neither in-game word frequency nor context had a significant impact on incidental learning gains.

Additional research investigated how much vocabulary could be learned by L2 English learners from gaming (Suprapas, 2021). The study explored the relationship between player/learner-oriented variables and vocabulary acquisition, highlighting gaming as a potential extramural incidental learning activity. A more recent study aimed to examine the impact of video games on vocabulary acquisition among Saudi university students (Alshabeb, 2024). The same research also explored the types of games deemed most effective and examined students’ perceptions of their use, with a recommendation that future research should investigate the long-term effects of video game-based learning and its influence on other language skills and explore different game types in various educational settings. Finally, research carried out while observing participants who played *Minecraft* (Mojang Studios, 2009) investigated how playing affects incidental vocabulary learning among young English as a Foreign Language (EFL) learners (Weisi & Hajizadeh, 2025). The study also considered gender differences in digital equity, and while there were no recommendations stemming from the study, the findings do contribute to the increasing research on digital games’ potential as an effective tool for promoting vocabulary development.

A commonly cited form of informal learning is language acquisition through living in a culture or community where that language is spoken. As a direct parallel of that scenario, it could be suggested that learning a language though an OWRPG is an easier and cheaper alternative to living and immersing oneself in a different country or community. Video games can serve as effective tools for incidental learning in linguistic contexts. However, despite the wealth of studies that focus on lexical acquisition, there is little research examining other subject areas and the impact of incidental learning. While one study by Champion and Hiriart (2024) examines the use of popular action role-playing games from the *Assassin’s Creed* (Ubisoft, 2007–2026) franchise to teach historical events and settings, to date no further research has been carried out around the use of commercial games as educational tools. It must also be noted that this research examines the “Discovery Tour” element of many *Assassin’s Creed* games, an element developed solely as an educational tool. As the official Assassin’s Creed website says:

“The Discovery Tour series ... let visitors explore Ancient Greece, Ancient Egypt, the Viking Age and Medieval Baghdad to learn more about their history and daily life ... students, teachers, non-gamers, and players can discover these eras at their own pace. In the games, they can embark on guided tours and stories curated by historians and experts.”

(Ubisoft, 2007–2026)

Discovery Tours provide a very different experience from the games themselves. They are passive experiences with no storyline or gameplay, no quest completion, combat, exploration, or “autonomy” to be had, and, importantly, any learning that occurs is intentional.

A similar study carried out by van Horssen et al. (2023) explored how video games could enhance learning in the higher education humanities classroom using two commercial digital games: *Sid Meier’s Civilization IV: Colonization* [Firaxis Games, 2008] and *GreedFall* (Spiders, 2019). Gameplay was paired with lecture content featuring moments in history that were also experienced in the games, and results indicated that students were better able to challenge historical narratives through reflexive engagement, and to question historical inaccuracies and utopian diversity. While reflexive critical thinking and analytic skills were honed, as with Champion and Hiriat’s study, any learning that was recorded was intentional.

A “Gaming for Good” report published in 2021 by ASK4 examined the role of gaming during the COVID-19 lockdown and asked higher education students to select up to five skills for which gaming had assisted in improving their cognitive ability. Respondents reported that gaming had enhanced their problem solving, strategic planning, concentration, critical thinking, and pattern recognition skills. Furthermore, students reported that gaming had improved their teamwork skills. These responses were consistent with the results of clinical studies by Kowal et al. (2018), which have shown that gamers have enhanced cognitive skills, including task switching and processing speed, enhanced visuo-motor coordination and problem-solving skills. A quantitative study carried out by Barr (2017) explored the impact of playing commercial video games on the development of graduate skills among undergraduate students in the arts and humanities. The study provided empirical evidence that commercial video games can be an effective tool for developing graduate skills, offering new opportunities for skills development in higher education institutions, challenging traditional perceptions of video games, and highlighting their potential as educational tools. Further research carried out by Barr (2022) builds on previous work by Gee (2003), Egenfeldt-Nielson (2007) and Squire (2013), who extolled the pedagogic potential of video games. This second study explored how commercial video games influence learning strategies among gamers, and identified these strategies as trial and error, consulting external sources, engaging in peer discussion, and applying conventions learned from one game to another. Barr (2022) suggests that there “may be scope for examining the role that video games may play in developing or even assessing these desired attributes in our students” and goes on to state that “games encourage and develop imaginative problem solving—this might, in fact, be unique to gaming. If this is the case, then video games may play a role in graduate development that higher education currently cannot fulfil.” However, it must be noted that in each of these studies, participants were aware that they were being asked to think intentionally of the skills they were learning while playing, rather than to think retrospectively of any skills they may have learned “accidentally.”

The finding that video games have the potential to improve students’ skills development over and above the acquisition of language is important in terms of further understanding of how students learn and may go some way to addressing the still-pervasive negative perception of gaming. To add weight to the argument that incidental learning takes place when playing OWRPGs, and that this form of knowledge acquisition is a powerful method for retaining knowledge via deep learning, it must also be noted that incidental learning links explicitly to established educational frameworks and principles of teaching and learning. It also connects with a number of semiotic domains, lending it credence over and above simply being a “happy accident,” or that any learning that has taken place is of little or no importance.

### 3.1. Incidental Learning and Semiotic Domains

To examine the importance of incidental learning that occurs in an OWRPG, one must first understand the concept of the semiotic domain: a structured context that uses multiple modalities such as language, images, symbols, or actions to create and convey meaning. Parallel to this, a semiotic domain such as oral or written language will also encompass a specific set of practices, knowledge, and values that are shared by a group of people who participate in that domain (Parmentier, 2016).

When individuals actively learn a new semiotic domain, they learn to experience the world in new ways, acquire the potential to join a new group of people with shared and distinctive social practices, and secure resources to prepare for future learning and problem solving, not only in this new domain, but also in related domains. Experiencing and exploring a new digital world; forming new affiliations with others, be this with other gamers within a multiplayer environment or with Non-Player Characters (NPCs) in a solo game; and preparing for future learning unite to form an ideal environment for active learning.

Therefore, OWRPGs provide digital spaces primed for higher education students to learn and to situate meanings through experiences in a complex semiotic domain, and a space for them to reflect on the process. Consider the gamer who, at the behest of a group of tormented villagers, uses logic or lateral thinking skills to unlock a haunted temple, goes on to defeat the ghostly (and ghastly) Arch Mage that guards the temple, and then returns to their home to display the sword they have been rewarded by the grateful villagers, all the while replaying the victory in their head but still wondering whether they could have dispatched the Arch Mage quicker, or without taking so much damage, and rethinking their future strategy. Furthermore, it can be argued that this active way of learning is better suited to a higher education setting, aligning as it does with the cognitive skills necessary to take part in post-compulsory education. Experiencing both the digital and semiotic domains provided by an open world can also help to prepare higher education students for the workplace, as many of the skills examined in this paper will attest.

### 3.2. Principles of Learning in OpenWorld Video Games

Gee (2003) examines the semiotic domains provided by games and proposes 36 principles of in-game learning that underpin how gamers learn while playing (see Appendix A). These principles are derived from his analysis of effective video game design and how it fosters engagement, motivation, and deep learning. While Gee states that these principles can be applied to formal education to improve pedagogical practices, it can also be argued that the practices inherent in many of Gee’s principles are fundamental to informal and incidental learning. Furthermore, it can be suggested that these areas complement one another: a learner who feels empowered will be better able to gain understanding, this elevated ability to learn will improve problem solving skills, and the player will be better equipped to navigate the social and cultural cues, behaviours, and expectations of the game world. This flags an opportunity for further research around the implications for neurodiverse students and gamers, who may feel safer learning and honing such skills in a digital environment over which they have complete autonomy. Furthermore, the highly structured nature of games and the immediate feedback they provide would be of particular use to such students, as would the reduced social pressure of games as compared to real-world interactions.

### 3.3. Inclusive and Accessible Environments, and Universal Design for Learning

Do OWRPGs contribute to better inclusivity within HE learning? The importance of inclusive practices in education is becoming increasingly recognised within the HE sector. A useful lens through which to evaluate the impact of OWRPGs on inclusivity is Universal Design for Learning (UDL), an educational framework that guides the creation of flexible learning environments (see Appendix B). It aims to remove barriers to learning, and in doing so, accommodate diverse learner needs from the outset. Developed by doctors David Rose and Anne Meyer from the Massachusetts-based Center for Applied Specialized Technology (CAST) in 1994, UDL looks to the neurosciences and to theories of progressive education (specifically constructivism), with reference to the work of Vygotsky and Cole (2018) and, less directly, Bloom (1956). The framework seeks to provide multiple options for how students can engage with, represent, and express what they know, thereby offering inclusive and accessible learning experiences for all, and is built on three principles:

1. *Multiple Means of Engagement:* to provide various ways for students to be interested, motivated, and connected to the material;
2. *Multiple Means of Representation:* to offer different ways for students to access and process information;
3. *Multiple Means of Action and Expression*: to give learners diverse ways to demonstrate their knowledge and skills (CAST, 2025).

OWRPGs offer digital environments in which to learn, with each of these three principles embedded explicitly. The open world offers *Multiple Means of Engagement*, *Representation and Action and Expression* through the choices made by the gamer from the outset, from their avatar’s appearance to their playstyle, their strategic approach, their moral compass, and how they navigate the in-game situations they encounter on their journey. Furthermore, the improvement of psychomotor skills, skills that require the brain to constantly process visual, auditory, and spatial stimuli while translating them into rapid, precise, and physical actions, was also apparent at the data analysis stage. Improved fine motor skills were mentioned repeatedly in participants’ responses. Such skills include hand–eye coordination, reaction times, precision of movement, and coordination.

(Health 360, 2023)

Of particular importance to neurodiverse gamers is the autonomy and pacing of OWRPGs, which allow players to choose their own objectives and tackle quests at their own pace without arbitrary real-life time constraints. This freedom prevents cognitive fatigue and overwhelm, as does the structured freedom provided by worlds which are vast but underpinned by coding and logic. The mix of open-ended exploration and defined game rules offers a perfect balance that appeals to both creativity and the need for organised systems. Linked to this, the steady stream of feedback such as discovering new areas, crafting items, or levelling up provides consistent bursts of dopamine (Greybeard, 2021).

Many RPGs allow players to experiment with social interactions, explore new identities, or build connections with welcoming, like-minded communities without the anxieties of real-life, unscripted social dynamics (ablegamers, 2022). It is this inclusiveness and accessibility that provides an environment in which incidental learning can take place. In doing so, players constantly encounter information and hone skills in informal and unintended ways: the hallmarks of incidental learning.

The level of inclusivity offered by UDL creates learning environments where all students, regardless of their background or preferred ways of learning, have an equal opportunity to succeed. Learner agency is provided as the environment shifts the focus to the student, providing them with choices and flexibility in their learning journey which foster motivation and engagement.

## 4. Discussion

**Figure 2:**
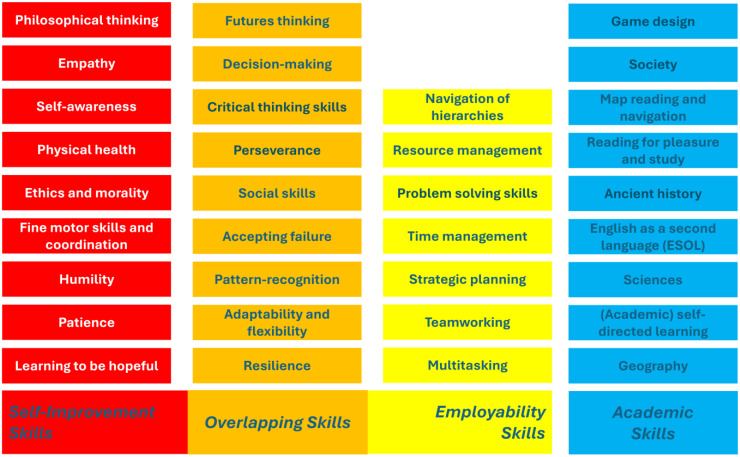
The Model of Incidental Learning in Open World Games (MILOWG), developed from the work of Fallows and Steven (2000), and highlighting skills developed by participants as an unintended consequence of playing OWRPGs. Self-improvement skills are represented in red, employability skills in yellow, and skills that belong to both categories represented in orange. Academic skills are represented in blue.

Students who play OWRPGs report experiencing incidental learning in a range of areas pertinent to the higher education sector. These areas can be categorised as *self-improvement skills*, *academic skills*, and *employability skills*. In total, 34 separate skills were noted by participants. Each of these skills were divided into the three categories, though it must be made clear that this was an activity based upon instinct and experience: there is no definitive set of criteria in which similar skills have previously been assigned. Many individual *self-improvement skills* and *employability skills* overlap as they contribute to both categories. Pertinently, employability skills form a fundamental part of the modern university’s curricula. Such skills *“include ... the retrieval and handling of information; communication and presentation; planning and problem solving; and social development and interaction”* (Fallows & Steven, 2000), many of which have been reported by participants and added to *(MILOWG)*, as seen in Figure 2, below:

Participants were keen to share their perceptions of incidental learning and appreciated that while their learning had been an unintended consequence of playing OWRPGs, these experiences had also opened a door to further formal study, the development of new interests, and new ways of navigating daily life. Skills and strategies learned in the digital environment were transferred into lived experiences where they were repeatedly practiced and honed, as exemplified by comments from two interview participants:

“I have noticed that my motor capabilities and ability to grasp more abstract concepts has definitely increased. I also tend to treat learning as ‘video-gamey quests’ to motivate myself. I think of learning as just an ability I need to ‘level up.’”

“Gaming has improved my problem-solving skills, decision-making, and adaptability. Games with deep lore have also expanded my interest in history, philosophy, and storytelling. OWRPGs encourage exploration and curiosity, which can translate to real world learning and creativity.”

All qualitative research is based on participants’ perceptions of the world around them. This being a CGT-based study, it must be noted that evidence of incidental learning comes from such perceptions. Furthermore, pre- or post-tests or objective skills assessments would mean looking for *intended* outcomes, thereby nullifying the “accidental” aspects that the research sought to discover. Many of the skills reported by participants may have been possessed before they started playing, and while the research design cannot determine whether the games actually *taught* new skills, it can be argued that OWRPGs provide a context for honing existing skills.

## 5. Limitations

It must be reiterated that these are the results of a single study where incidental learning is but one unintended consequence that occurs as a result of playing OWRPGs. Implications are that language acquisition is *not* the only area where such learning can occur; indeed, this research has demonstrated that other academic subject areas see incidental learning occurring. Furthermore, there is evidence that when higher education students play OWRPGs, they demonstrate ongoing self-improvement and development of employability skills.

However, it must be noted that the demographics of the study are limited as 73% of participants were male, and all studied at universities based in the United Kingdom. It is recommended that future research investigate in more depth the experiences of women and non-binary gamers, non-student populations, different age groups and gamers from other cultural backgrounds. It is also important to note that observations made are from self-reported, perceived impacts from participants, not objective measures. To show objective impacts, a more tailored intervention is suggested, including pre- and post-tests.

Linked to this, all participants were interviewed around their experiences playing a single genre of video game that, while allowing players the freedom to play in any style, is still but one genre. Therefore, it must be stated that, were the same research carried out using different genres of video game, it would be logical to assume that the results could be different. Returning to the possible issues of interviewing higher education students only, it must be noted that this is a socioeconomic group that is particularly responsive and well versed to learning. Therefore, this research should be seen as a starting point for further, broader research examining the range and impact of incidental learning in gamers of different ages, of different socioeconomic backgrounds, playing alternative genres, and accessing different devices. Finally, to substantiate and test the validity of this unintended consequence, it would be useful to return to participants for further semi-structured interviews.

## 6. Recommendations and Conclusions

The primary objective of this paper is to highlight skills that United Kingdom-based higher education students feel they can hone through incidental learning, and as a byproduct of playing OWRPGs. Key findings indicate that many skills vital to the progression of such students can be learned or improved through incidental learning. Furthermore, these skills can be categorised as *self-improvement skills, employability skills,* and *academic skills.*

Yet, despite the ever-increasing number of students studying at post-compulsory levels who regularly play OWRPGs, a school of thought remains that posits gaming as, at best, a waste of time and, at worst, a harmful and addictive endeavour. Were teaching staff made aware of the power of commercial OWRPGs to generate incidental learning in a number of academic subject areas, it could be suggested that there may be some reduction in negative attitudes towards gaming. Meanwhile, with improvements in skills such as time management, self-awareness, and decision-making, many student gamers may feel less guilt around their own gaming habits.

The implications of these findings are certainly thought-provoking. While a number of studies have examined lexical acquisition as one form of incidental learning that can occur when playing video games, the dearth of studies examining other academic or skills areas highlights a gap in the research. The new data presented here contributes to existing research by adding weight to the argument that incidental learning can take place as a result of playing OWRPGs, but also acknowledges limitations such as this being a one-off rather than a longitudinal study, and that none of the skills participants talked about in their interviews have been tested to quantify the impact or amount of learning that took place. As mentioned above, it would also be worth examining gaming genres other than OWRPGs, as studying a single genre limits the scope of the research. Furthermore, this study goes some way toward disproving the notion that all unintended consequences are negative, highlighting, as it does, that there can be “happy accidents” as well as dire unintended outcomes.

In conclusion, the power and reach of incidental learning through playing OWRPGs makes for a largely untapped potential that demands more attention and rigorous research. With almost 86% of higher education students in the United Kingdom (ASK4, 2021) playing video games, gaming is no longer a niche hobby or interest. Therefore, it is suggested that further research look at occurrences of incidental learning through other video game genres and examine more closely the impact of self-improvement, academic, and employability skills mentioned in this paper, and how such skills could, theoretically, improve student outcomes.

## Data Availability

The raw data supporting the conclusions of this article will be made available by the author on request.

## Appendix A. Gee’s 36 Learning Principles of Learning in Video Games

1. **Active, Critical Learning Principle**
All aspects of the learning environment (including the ways in which the semiotic domain is designed and presented) are set up to encourage active and critical, not passive, learning.
Links to all self-improvement, employability, and academic skills
2. **Design Principle**
Learning about and coming to appreciate design and design principles is core to the learning experience.
Links to all self-improvement, employability, and academic skills
3. **Semiotic Principle**
Learning about and coming to appreciate interrelations within and across multiple sign systems (images, words, actions, symbols, artefacts, etc.) as a complex system is core to the learning experience.
Links to all academic skills
4. **Semiotic Domain Principle**
Learning involves mastering, at some level, semiotic domains, and being able to participate, at some level, in the affinity groups connected to them.
**Links to all self-improvement, employability, and academic skills**
5. **Meta-Level Thinking about Semiotic Domain Principle**
Learning involves active and critical thinking about the relationships of the semiotic domain being learned to other semiotic domains.
Links to all self-improvement, employability, and academic skills
6. **“Psychosocial Moratorium” Principle**
Learners can take risks in a space where real-world consequences are lowered.
Links to all self-improvement, employability, and academic skills
7. **Committed Learning Principle**
Learners participate in an extended engagement (lots of effort and practice) as extensions of their real-world identities in relation to a virtual identity to which they feel some commitment and a virtual world they find compelling.
Links to all self-improvement, employability, and academic skills
8. **Identity Principle**
Learning involves taking on and playing with identities in such a way that the learner has real choices (in developing the virtual identity) and ample opportunity to meditate on the relationship between new identities and old ones. There is a tripartite play of identities as learners relate, and reflect on, their multiple real-world identities, a virtual identity, and a projective identity.
Links to all self-improvement and employability skills
9. **Self-Knowledge Principle**
The virtual world is constructed in such a way that learners learn not only about the domain, but also about themselves and their current and potential capacities.
Links to all self-improvement and employability skills
10. **Amplification and Input Principle**
For little input, learners get a lot of output.
Links to self-improvement and employability skills, with especially strong link to academic skills
11. **Achievement Principle**
For learners of all levels of skill, there are intrinsic rewards from the beginning, customised to each learner’s level, effort, and growing mastery and signalling the learners’ ongoing achievements.
Links to all self-improvement, employability, and academic skills
12. **Practice Principle**
Learners get lots of practice in a context where the practice is not boring (i.e., in a virtual world that is compelling to learners on their own terms and where learners experience ongoing success). They spend lot of time on tasks.
Links to all self-improvement, employability, and academic skills
13. **Ongoing Learning Principle**
The distinction between learner and master is vague, since learners, thanks to the operation of the “regime of competence” principle must, at higher and higher levels, undo their routinised mastery to adapt to new or changed conditions. There are cycles of new learning, automatisation, undoing automatisation, and new reorganised automatisation.
Especially strong links to self-improvement and employability skills, with weaker links to academic skills
14. **“Regime of Competence” Principle**
The learner gets ample opportunity to operate within, but at the outer edge of, his or her resources, so that at those points things are felt as challenging but not “undoable.”
Links to all self-improvement, employability, and academic skills
15. **Probing Principle**
Learning is a cycle of probing the world (doing something); reflecting in and on this action and on this basis, forming a hypothesis; re-probing the world to test this hypothesis; and then accepting or rethinking this hypothesis.
Links to all self-improvement and employability skills
16. **Multiple Routes Principle**
There are multiple ways to make progress or move ahead. This allows learners to make choices, relying on their own strength and styles of learning and problem solving, while also exploring alternative styles.
Links to all self-improvement, employability, and academic skills
17. **Situated Meaning Principle**
The meanings of signs (words, actions, objects, artefacts, symbols, texts, etc.) are situated in embodied experience. Meanings are not general or decontextualised. Whatever generality meanings come to have is discovered bottom up via embodied experiences.
Links to all self-improvement, employability, and academic skills
18. **Text Principle**
Texts are understood purely verbally (i.e., in terms of the definitions of the words in the text and the intertextual relationships to each other) but are understood in terms of embodied experiences. Learners move back and forth between texts and embodied experiences. More purely verbal understanding (reading texts apart from embodied action) comes only when learners have had enough embodied experience in the domain and ample experiences with similar texts.
Links to all academic skills
19. **Intertextual Principle**
The learner understands texts as a genre of related texts and understands any one such in relation to others in the family, but only after having achieved embodied understandings of some texts. Understanding a group of texts as a family (genre) of texts is a large part of what helps the learner make sense of such texts.
Links to all academic skills
20. **Multimodal Principle**
Meaning and knowledge are built through various modalities (images, texts, symbols, interactions, abstract design, sounds, etc.), not just words.
Links to all self-improvement, employability, and academic skills
21. **“Material Intelligence” Principle**
Thinking, problem solving, and knowledge are stored in material objects and the environment. This frees learners to engage their minds with other things while combining the results of their own thinking with the knowledge stored in material objects and the environment to achieve yet more powerful effects.
**Links to all self-improvement, employability, and academic skills**
22. **Intuitive Knowledge Principle**
Intuitive or tacit knowledge built up in repeated practice and experience, often in association with an affinity group, counts for a great deal and is honoured. Not just verbal and conscious knowledge is rewarded.
Links to all self-improvement, employability, and academic skills
23. **Subset Principle**
Learning even at its start takes place in a (simplified) subset of the real domain.
Links to all self-improvement, employability, and academic skills
24. **Incremental Principle**
Learning situations are ordered in the early stages so that earlier cases lead to generalisations that are fruitful for later cases. When learners face more complex cases later, the learning space (the number and type of guesses the learner can make) is constrained by the sorts of fruitful patterns or generalisations the learner found earlier.
Links to all self-improvement, employability, and academic skills
25. **Concentrated Sample Principle**
The learner sees, especially early on, many more instances of fundamental signs and actions than would be the case in a less controlled sample. Fundamental signs and actions are concentrated in the early stages so that learners get to practice them often and learn them well.
Links to all self-improvement, employability, and academic skills
26. **Bottom-Up Basic Skills Principle**
Basic skills are not learned in isolation or out of context; rather, what counts as a basic skill is discovered bottom-up by engaging in more and more of the game/domain or game/domains like it. Basic skills are genre elements of a given type of game/domain.
Links to all self-improvement, employability, and academic skills
27. **Explicit Information On-Demand and Just-in-Time Principle**
The learner is given explicit information both on demand and just in time, when the learner needs it or just at the point where the information can best be understood and used in practice.
Links to all self-improvement, employability, and, to a greater extent, academic skills
28. **Discovery Principle**
Overt telling is kept to a well-thought-out minimum, allowing ample opportunity for the learner to experiment and make discoveries.
Links to all self-improvement, employability, and, to a lesser extent, academic skills
29. **Transfer Principle**
Learners are given ample opportunity to practice and support for transferring what they have learned earlier to later problems, including problems that require adapting and transforming that earlier learning.
Links to all self-improvement and employability skills
30. **Cultural Models about the World Principle**
Learning is set up in such a way that learners come to think consciously and reflectively about some of their cultural models regarding the world, without denigration of their identities, abilities, or social affiliations, and juxtapose them to new models that may conflict with or otherwise relate to them in various ways.
Links to all self-improvement and employability skills
31. **Cultural Models about Learning Principle**
Learning is set up in such a way that learners come to think consciously and reflectively about their cultural models of learning and themselves as learners, without denigration of their identities, abilities, or social affiliations, and juxtapose them to new models about this domain.
Links to all self-improvement and employability skills
32. **Cultural Models about Semiotic Domain Principle**
Learning is set up in such a way that learners come to think consciously and reflectively about their cultural models about a particular semiotic domain they are learning, without denigration of their identities, abilities, or social affiliations, and juxtapose them to new models about this domain.
Links to all self-improvement and employability skills
33. **Distributed Principle**
*Meaning/knowledge is distributed across the learner, objects, tools, symbols, technologies, and the environment.*
Links to all self-improvement, employability, and academic skills
34. **Dispersed Principle**
*Meaning/knowledge is dispersed in the sense that the learner shares it with others outside the domain/game, some of whom the learner may rarely or never see face-to-face.*
Links to all self-improvement, employability, and academic skills
35. **Affinity Group Principle**
Learners constitute an “affinity group,” that is, a group that is bonded primarily through shared endeavours, goals, and practices and not shared race, gender, nation, ethnicity, or culture.
Links to all self-improvement, employability, and to a lesser extent, academic skills
36. **Insider Principle**
The learner is an “insider,” “teacher,” and “producer,” (not just a “consumer”) able to customise the learning experience and domain/game from the beginning and throughout the experience.
Links to all self-improvement, employability, and academic skills
